# Trapped in Silence: Severe Catatonia From Anti-N-Methyl-D-Aspartate (NMDA) Receptor Encephalitis in a Neurofibromatosis Type 1 Patient With Craniopharyngioma

**DOI:** 10.7759/cureus.98336

**Published:** 2025-12-02

**Authors:** Kirubel Z Gebreselassie, Tizita Negash, Pablo Andres Bravo, Wayne Chiu

**Affiliations:** 1 Internal Medicine, Northside Hospital, Atlanta, USA; 2 Epidemiology and Public Health, Emory University Rollins School of Public Health, Atlanta, USA; 3 Neurology, Northside Hospital, Atlanta, USA

**Keywords:** anti nmda receptor encephalitis, craniopharyngioma surgery, neurofibromatosis 1 (nf1), refractory catatonia, ventriculoperitoneal (vp) shunt

## Abstract

We report a challenging case of anti-N-methyl-D-aspartate receptor (NMDAR) encephalitis in a 36-year-old female with neurofibromatosis type I (NF1) and a history of craniopharyngioma. Despite a functional ventriculoperitoneal (VP) shunt and negative infectious workup, she developed severe catatonia and neuropsychiatric symptoms, requiring intensive immunotherapy and psychiatric care. Diagnostic challenges arose from overlapping manifestations of neurofibromatosis I, recent central nervous system (CNS) procedures, and autoimmune encephalitis mimicking a psychiatric illness. Catatonia masked early treatment response, complicating clinical assessment. Her condition improved following coordinated multidisciplinary care, emphasizing the critical need for timely recognition and management of catatonia in autoimmune encephalitis, particularly in patients with neurogenetic disorders and recent neurosurgical history.

## Introduction

Anti-N-methyl-D-aspartate receptor (NMDAR) encephalitis is a rare autoimmune disorder predominantly affecting young adults, particularly females, and often presents with psychiatric symptoms that can mimic infectious or primary psychiatric conditions. While commonly linked to ovarian teratomas, around 40% of cases occur without tumors, frequently following infections or surgeries [1,2]. This report is distinctive as it documents anti-NMDAR encephalitis in the absence of tumor association, occurring in a patient with neurofibromatosis type 1 (NF1) and a prior craniopharyngioma treated surgically. NF1, an autosomal dominant disorder caused by *NF1 *gene mutations on chromosome 17q11.2, is typically associated with cutaneous and ocular findings but also includes central nervous system (CNS) involvement, such as cognitive and attention deficits [3,4]. These overlapping features can complicate the diagnostic process when autoimmune encephalitis is suspected [4]. Catatonia, seen in 50-80% of anti-NMDAR encephalitis cases [5], presents with mutism, stupor, and rigidity, and may be misdiagnosed as a psychiatric illness. It results from NMDAR hypofunction and often requires immunotherapy, sometimes with benzodiazepines or electroconvulsive therapy (ECT) [6].

## Case presentation

**Figure 1:**
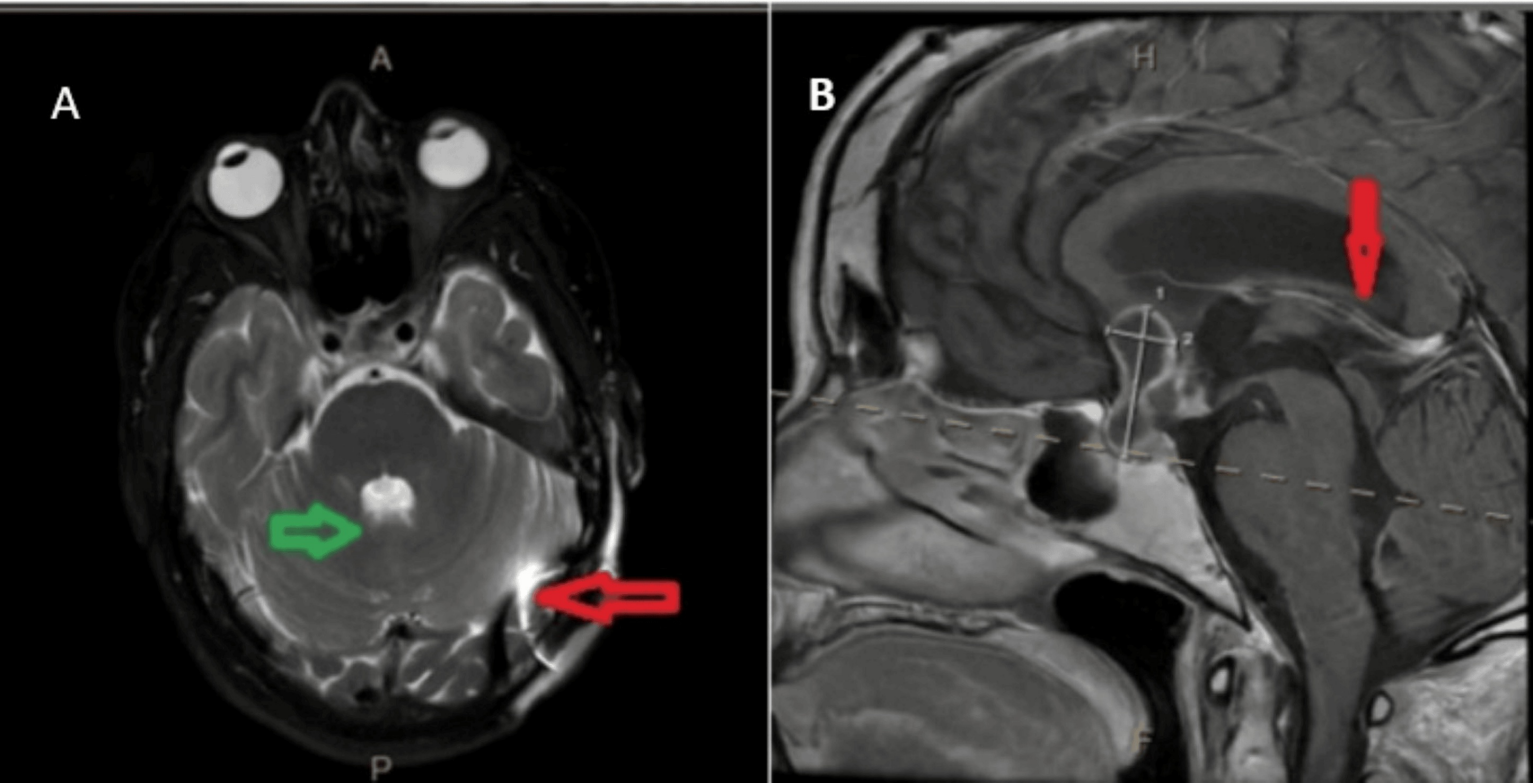
MRI brain with and without contrast A. Axial T2-weighted MRI. B Sagittal section T1 MRI. Green arrow: stable peripherally enhancing cystic sellar/suprasellar lesion consistent with a history of craniopharyngioma. Red arrow: indwelling left occipital ventriculostomy catheter intact, stable size and configuration of the ventricular system MRI: magnetic resonance imaging

A 36-year-old female with NF1 and a history of craniopharyngioma resection (May 2024) (Figure 1) presented with subacute neuropsychiatric symptoms. She had previously undergone ventriculoperitoneal (VP) shunt placement in March 2024 for obstructive hydrocephalus and had completed 30 sessions of cranial radiation therapy. Until early 2025, she remained neurologically intact, independent, and employed in two jobs.

**Figure 2:**
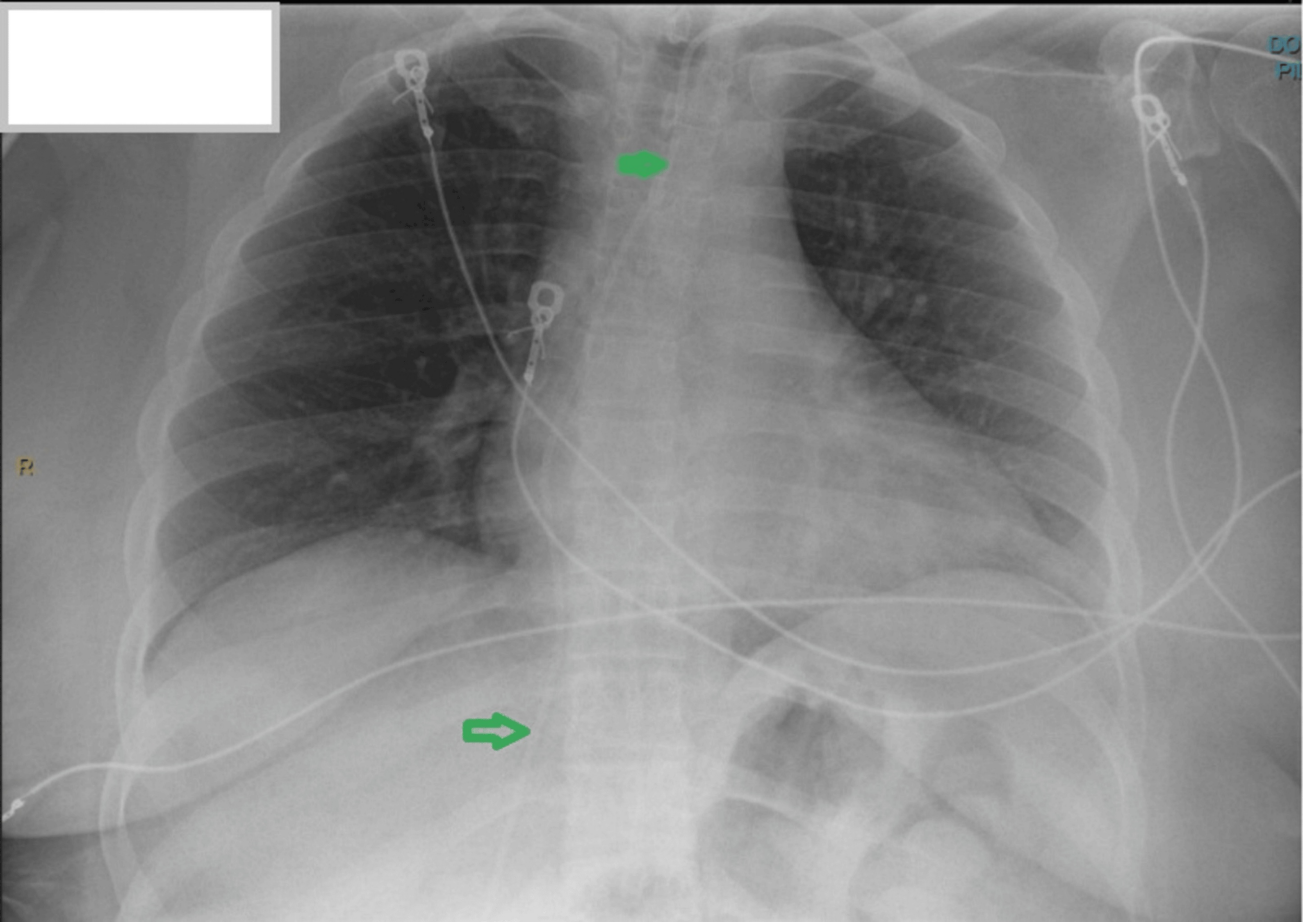
Ventriculoperitoneal shunt series Green arrow: intact ventriculoperitoneal shunt, which terminates at the right lower quadrant

In February 2025, she developed memory issues, confusion, and stuttering approximately 14 months after a left occipital VP shunt placement and left frontal craniotomy for transventricular resection of the tumor. Initial outpatient evaluation, including neuroimaging and shunt assessment, was unremarkable. Lamotrigine was started on February 14 for presumed seizure-related symptoms. Her condition progressively worsened, leading to hospitalization from February 15-17 for evaluation and management of agitation and confusion. MRI, electroencephalogram, and shunt imaging (Figure 2) showed no significant changes, and infectious and metabolic evaluations were unremarkable. She was discharged on lamotrigine and olanzapine due to persistent short-term memory deficits and intermittent episodes of agitation.

The patient's condition continued to deteriorate, and she was admitted to our institution on February 20 with worsening confusion accompanied by new-onset involuntary movements. She displayed agitation, inappropriate gestures, and sudden mutism. At baseline, she had been cognitively and socially intact.

During this admission, repeat imaging and EEG remained non-revealing. CT of the chest, abdomen, and pelvis showed no malignancy. Lumbar puncture on February 26 revealed lymphocytic pleocytosis and elevated CSF protein (64 mg/dL) (Table 1); infectious and metabolic screens were negative. Although this CSF profile is consistent with autoimmune encephalitis, the diagnosis was ultimately confirmed by the detection of anti-NMDAR antibodies in both serum and CSF (Table 2).

**Table 1:** CSF analysis with autoimmune and paraneoplastic panel (1/2) CSF: cerebrospinal fluid; AMPAR-R1 Ab, CSF: α-amino-3-hydroxy-5-methyl-4-isoxazolepropionic acid receptor subtype 1 antibody; AMPAR-R2 Ab, CSF: α-amino-3-hydroxy-5-methyl-4-isoxazolepropionic acid receptor subtype 2 antibody; AGNA-1, CSF: anti-glial nuclear antibody type 1; ANNA-1, CSF: anti-neuronal nuclear antibody type 1 (also known as Hu antibody); ANNA-2, CSF: anti-neuronal nuclear antibody type 2 (also known as Ri antibody); CRMP-5-IgG CBA CSF: collapsin response mediator protein 5 IgG (cell-based assay); CASPR2-IgG CBA CSF: contactin-associated protein-like 2 IgG (cell-based assay); DPPX-IgG CBA CSF: dipeptidyl-peptidase-like protein 6 IgG (cell-based assay); GABA-B-R Ab, CSF: gamma-aminobutyric acid type B receptor antibody

Test	Result	Reference range	Unit	Status/notes
Glucose, CSF	63 *	40 – 70	mmol/L	Slightly elevated (upper-normal)
Protein, CSF	64 * (H)	15 – 45	g/L	High
CSF culture	Negative	–	–	No growth
Herpes simplex 1	Not detected (RTPCR -)	Not detected (RTPCR -)	–	Negative
Herpes simplex 2	Not detected (RTPCR -)	Not detected (RTPCR -)	–	Negative
Escherichia coli K1	Negative	Negative	–	–
Haemophilus influenzae	Negative	Negative	–	–
Listeria monocytogenes	Negative	Negative	–	–
Neisseria meningitidis	Negative	Negative	–	–
Streptococcus agalactiae	Negative	Negative	–	–
Streptococcus pneumoniae	Negative	Negative	–	–
Cytomegalovirus	Negative	Negative	–	–
Cryptococcus neoformans/gattii	Negative	Negative	–	–
Enterovirus	Negative	Negative	–	–
Herpes simplex virus 1	Negative	Negative	–	–
Herpes simplex virus 2	Negative	Negative	–	–
Human herpes virus 6	Negative	Negative	–	–
Human parechovirus	Negative	Negative	–	–
Varicella zoster virus	Negative	Negative	–	–
Angiotensin-converting enzyme, CSF	1.4	0.0 – 2.5	U/L	Within range
AMPA-R Ab CBA, CSF	Negative	Negative	–	–
AGNA-1, CSF	Negative	Negative	–	–
Amphiphysin Ab, CSF	Negative	Negative	–	–
ANNA-1, CSF	Negative	Negative	–	–
ANNA-2, CSF	Negative	Negative	–	–
ANNA-3, CSF	Negative	Negative	–	–
CASPR2-IgG CBA, CSF	Negative	Negative	–	–
CRMP-5-IgG, CSF	Negative	Negative	–	–
DPPX Ab CBA, CSF	Negative	Negative	–	–
GABA-B-R Ab CBA, CSF	Negative	Negative	–	–

**Table 2:** CSF analysis with autoimmune and paraneoplastic panel (2/2) CSF: cerebrospinal fluid; NMDA-R CBA Ab: N-methyl-D-aspartate receptor antibody test; GAD65: glutamic acid decarboxylase 65; GFAP IFA: glial fibrillary acidic protein (an intermediate filament antibody); IgLON5: immunoglobulin-like cell adhesion molecule; (LGI1) Ab: leucine-rich glioma-inactivated 1 antibody; DNER: delta and notch-like epidermal growth factor-related receptor; mGluR1: metabotropic glutamate receptor 1; NIF: neuronal intermediate filament; PCA: Purkinje cytoplasmic antibody; PDE10A: phosphodiesterase 10A; TRIM46: tripartite motif-containing protein 46

Test	Result	Reference range	SI unit	Notes/status
GAD65 Ab assay, CSF	0.00 *	≤ 0.02	nmol/L	Negative
GFAP IFA, CSF	Negative *	Negative	—	
IgLONS CBA, CSF	Negative *	Negative	—	
LGI1-IgG CBA, CSF	Negative *	Negative	—	
Neurochondrin IFA, CSF	Negative *	Negative	—	
mGluR1 Ab IFA, CSF	Negative *	Negative	—	
NIF IFA, CSF	Negative *	Negative	—	
NMDAR Ab CBA, CSF	Positive (H)	Negative	—	Abnormal
NMDAR Ab Titer, CSF	Positive 1:8 (H)	<1:2	—	Abnormal
PCA-Tr, CSF	Negative *	Negative	—	
PCA-1, CSF	Negative *	Negative	—	
PCA-2, CSF	Negative *	Negative	—	
PDE10A Ab IFA, CSF	Negative *	Negative	—	
TRIM46 Ab IFA, CSF	Negative *	Negative	—	
Septin-7 IFA, CSF	Negative *	Negative	—	
CSF tube number	4	—	—	
CSF color	Colorless *	—	—	Normal
CSF appearance	Clear	—	—	Normal
CSF RBC	39	0	Cells/µL	Slightly elevated
CSF nucleated cells	11 (H)	0 – 5	Cells/µL	Elevated
CSF neutrophils	0	0 – 6	%	Normal
CSF lymphocytes	95 (H)	40 – 80	%	Elevated
CSF monocytes/macrophages	5 (L)	15 – 45	%	Low
CSF total cells counted	100	—	Cells	

The patient was treated with a five-day intravenous immunoglobulin (IVIG) course, followed by high-dose methylprednisolone (1 g/day for five days) and rituximab 1000 mg (March 13). Olanzapine 5 mg SL BID reduced agitation, but mutism and emotional lability persisted. Five sessions of plasmapheresis were initiated in April, with two additional sessions for refractory symptoms. A second rituximab dose was given in May.

One of the most challenging features of her clinical course was the manifestation of severe catatonia, which appeared to mask improvements from immunotherapy. Despite immunologic treatment, she remained largely mute and physically unresponsive. Psychiatry was consulted, and she was diagnosed with catatonia secondary to anti-NMDAR encephalitis. Lorazepam was initiated at 1 mg three times daily and later titrated to 2 mg TID, resulting in gradual improvement in responsiveness and reduction of psychomotor inhibition. The catatonic symptoms had significantly blunted her clinical assessment and delayed visible progress, underscoring the need for early psychiatric intervention in similar cases.

By the time of discharge in April 2025, the patient was able to follow commands briskly and communicate using short sentences. Although her short-term memory remained impaired, agitation and behavioral symptoms had improved. Ativan and olanzapine were tapered to the lowest effective doses, with plans for complete weaning in the outpatient setting. She was continued on lamotrigine and lacosamide for seizures and discharged after a two-month hospital stay. Ongoing follow-up was arranged with autoimmune neurology and psychiatric services, with plans for continued immunosuppression using weekly Solu-Medrol infusions over six weeks.

## Discussion

This case illustrates the clinical complexity of diagnosing and managing anti-NMDAR encephalitis in a patient with NF1 and recent treatment for craniopharyngioma, including VP shunt placement and cranial irradiation. While anti-NMDAR encephalitis is a well-characterized autoimmune condition most frequently associated with ovarian teratomas, up to 40% of cases occur without identifiable tumors. These instances may be triggered by CNS infections, surgeries, or immune dysregulation.

The development of anti-NMDAR encephalitis in the setting of neurocutaneous disorders like NF1 and recent CNS interventions such as craniopharyngioma resection reflects a multifactorial risk environment involving both genetic susceptibility and iatrogenic triggers [7]. Although NF1 is not typically associated with anti-NMDAR encephalitis, mounting evidence suggests that patients with NF1 may have altered immune function. Studies have demonstrated T and B cell dysregulation in NF1 murine models [8], and patients with NF1 are reportedly more susceptible to autoimmune conditions such as [9]. These findings raise the possibility that NF1 may confer baseline vulnerability to CNS-directed autoimmunity, including anti-NMDAR encephalitis [10]. Although craniopharyngiomas themselves do not express NMDA receptors, the extensive CNS manipulation required for tumor resection, VP shunt placement, and radiation therapy may have served as a triggering factor [11]. These interventions likely disrupted the blood-brain barrier, potentially exposing neuronal antigens to the peripheral immune system and contributing to the production of pathogenic autoantibodies.

Our patient’s disease course underscores how the intersection of NF1-associated immune dysregulation and iatrogenic CNS exposure may create a permissive environment for autoimmune encephalitis. The absence of a paraneoplastic source further emphasizes the importance of non-tumor triggers in susceptible individuals. The clinical features observed in our patient, ranging from behavioral dysregulation and mutism to seizures and autonomic instability, fit the classic spectrum of anti-NMDAR encephalitis. A particularly challenging feature was the co-occurrence of catatonia, which appeared to mask the response to immunosuppressive therapy and required targeted psychiatric management with lorazepam. Notably, the resolution of catatonic symptoms helped reveal the underlying therapeutic response to immunotherapy.

Moreover, catatonia, a neuropsychiatric syndrome characterized by mutism, stupor, and posturing, is increasingly recognized in anti-NMDAR encephalitis. A prospective study by Espinola-Nadurille et al. reported a 70% prevalence of catatonia among confirmed cases, where it often co-occurred with agitation, hallucinations, and EEG abnormalities [5]. These findings underscore that catatonia is not merely a psychiatric curiosity but a frequent and diagnostically significant marker of autoimmune encephalitis, particularly when refractory to standard antipsychotics.

Furthermore, Wadi and Mandge reported cases of malignant catatonia secondary to anti-NMDAR encephalitis that necessitated ICU-level care, with significant clinical improvement observed only following ECT [12]. This emphasizes that treatment-resistant catatonia may necessitate both immunomodulatory and psychiatric interventions, reinforcing the value of a multidisciplinary approach in managing complex cases. Given this complexity, clinicians should maintain a high index of suspicion for anti-NMDAR encephalitis in patients with neurogenetic syndromes or recent neurosurgical histories who present with new-onset psychiatric or cognitive symptoms. Early lumbar puncture and antibody testing are essential. Equally important is the prompt recognition and treatment of catatonia, which may coexist and obscure the assessment of treatment efficacy.

Limitations of the study

As a single case report, this study cannot establish a definitive causal link between NF1-related immune dysregulation, prior CNS surgery, radiation therapy, and autoimmune predisposition. Our goal was to highlight a potential association that may warrant further investigation, and we acknowledge the inherent limitations in drawing firm conclusions.

## Conclusions

This report underscores the diagnostic complexity of anti-NMDAR encephalitis in patients with underlying neurological conditions like NF1 and recent CNS interventions. Anti-NMDAR encephalitis can present as a psychiatric illness and can easily be missed, delaying treatment initiation. Catatonia, a frequent but often overlooked manifestation, can obscure clinical assessment and delay appropriate treatment. Prompt immunotherapy combined with psychiatric intervention led to significant recovery in our patient. The potential interplay between NF1-related immune dysregulation and iatrogenic CNS trauma suggests a permissive environment for autoantibody production. Further research is needed to assess the prevalence of autoimmune encephalitis in NF1 and post-neurosurgical populations, clarify immune mechanisms, and develop early screening strategies. Recognizing the convergence of genetic, surgical, and immunologic factors is crucial for timely diagnosis and improved outcomes.
